# Establishment and validation of an evaluation system for hospital infection prevention and control courses: a study protocol using the Delphi method and analytic hierarchy process

**DOI:** 10.3389/fpubh.2025.1645429

**Published:** 2025-09-19

**Authors:** Jing Mu, Qiyuan Huang, Jiajia Tu, Fang Liu

**Affiliations:** ^1^Department of Orthopedics, The Affiliated Suqian Hospital of Xuzhou Medical University, Suqian, China; ^2^School of Nursing, Wenzhou Medical University, Wenzhou, China; ^3^Department of Nursing, Second People’s Hospital of Yichang City, Yichang, China; ^4^School of Nursing, Xuzhou Medical University, Xuzhou, China

**Keywords:** Delphi method, hospital infection prevention and control courses, CIPP model, course evaluation, education

## Abstract

**Background:**

The evaluation of hospital infection prevention and control (HIPC) courses holds significant importance in guaranteeing the quality. Regrettably, there is currently no specific evaluation tool available in China for this purpose. This study aims to develop a comprehensive system to evaluate the HIPC courses in China.

**Methods:**

The authors developed an initial draft for a curriculum evaluation system, based on the context, input, process, and product model, a literature review, and semi-structured interviews with 23 participants. Subsequently, an evaluation system was established via two rounds of Delphi surveys involving 18 experts from 7 A-grade tertiary hospitals and 11 higher medical education institutions across China. The validity of the evaluation system was further confirmed using the Analytic Hierarchy Process (AHP), ensuring a comprehensive assessment of the established framework.

**Results:**

After two rounds of correspondence, the evaluation index system includes four first-level indicators, 13 second-level indicators, and 52 third-level indicators. The expert authority coefficients for these rounds were 0.869 and 0.887, respectively, indicating a high level of expertise among the participating experts. Additionally, the Kendall’s W of each index are, respectively, was 0.153 ~ 0.162 and 0.168 ~ 0.175 (*p* < 0.05). The consistency test was conducted using the AHP for all judgment matrices, with a consistency ratio (CR) for all levels of indicators < 0.10, indicating good consistency in the weight settings. Among the four first-level indicators, the weight of the “Course Process” was the highest (0.5857), followed by the “Course Product” (0.2389), while the weights for the “Course Context” and “Course Input” were the same (0.0877).

**Conclusion:**

The evaluation system for the hospital infection prevention and control courses is CIPP-oriented, comprehensive, and reliable. It offers a practical framework for comprehensively assessing the teaching effectiveness of the courses and enhancing educational quality.

## 1 Introduction

Hospital Infection Prevention and Control (HIPC) courses are an emerging interdisciplinary discipline in modern medical education, including research on the occurrence, development, control, and management of HAIs (1, 2). Education in HIPC courses is an important assurance of the quality of training in the healthcare sector (3). By studying HIPC, medical students can master professional knowledge such as exposure mechanisms and risk prevention, thereby contributing to the creation of a safe and high-quality clinical medical work environment (2). The World Health Organisation (WHO) reports that Hospital-acquired Infections (HAIs) have become one of the most common adverse events in the healthcare system (4). Effective education in HIPC can regulate healthcare workers’ practice patterns and reduce the likelihood of HAIs. Therefore, the provision of quality education in HIPC is critical to the reduction of medical errors and the saving of costs of healthcare resources (5). Driven by the medical education context of “Healthy China,” the development of talents and the improvement of teaching skills are required due to the reform of higher medical education in university teaching institutions (6). Improving the quality of HIPC courses in higher education facilitates the development of faculty teaching skills and student learning.

In order to improve the infection control skills of medical students, university teaching institutions worldwide are gradually improving their systems for teaching HIPC courses. Previous studies have shown that institutions of higher learning in Europe and the United States focus on the integration of interdisciplinary paradigms in HIPC education to develop students’ infection control skills and knowledge of clinical microbiology as the core of the program and provide HIPC courses for undergraduate students in a wide range of disciplines (7). In addition, the teaching methods of HIPC courses are being technologically upgraded, driven by digital technology. Wolf et al. (8) developed an interactive multimedia infection control teaching module that teaches and interacts with medical students through case studies and animated presentations, based on the teaching content of pathophysiology and health assessment. Masson et al. (9) applied virtual reality to the infection control teaching of safe operating theatre practices to medical students to improve their infection control literacy through simulated demonstrations of infection control precautions and correct behaviors. Compared with the more mature HIPC teaching system in Western countries, the development of HIPC courses in China is lagging. Existing research shows that Chinese higher medical institutions are actively exploring a new model of HIPC education system in the era of “digital China” (10, 11). However, the absence of a scientifically grounded tool to evaluate HIPC teaching quality in China hinders the development of high-quality HIPC education.

This study adopts the Context, Input, Process, Product (CIPP) model developed by Daniel Stufflebeam as its theoretical guide. This model enables clear alignment between the evaluation logic and the characteristics of the target curriculum (12, 13). Based on the CIPP theory, the model evaluation system constructed in this study can cover the entire life cycle of the HIPC curriculum, thereby realizing the organic integration of curriculum, evaluation, teaching, and learning. Existing studies have applied CIPP in medical education to evaluate curricula and promote iterative quality improvement, which indicates that this evaluation model is applicable to the evaluation of complex medical curricula (14–16). Xiao et al. (17) constructed an evaluation system for Virtual Teaching and Research Offices (VTROs) in medical education under the guidance of the CIPP model, and the results showed that this framework features comprehensive coverage and operability. Zhao et al. (18) established a quality evaluation system for public health practical teaching based on the CIPP model. This system is reliable and highly adaptable, and can effectively identify the strengths and weaknesses of teaching quality. The CIPP model can provide a systematic and comprehensive perspective and is applicable to the evaluation of complex and ever-changing medical curricula, which lays a solid theoretical foundation for this study. Considering that there is currently a lack of uniformly recognized HIPC curriculum evaluation tools in China, and multiple dimensions and hierarchical levels characterize the indicators, this study adopts the Delphi technique to collect expert consensus anonymously and enhance content validity. Additionally, it uses the Analytic Hierarchy Process (AHP) to obtain repeatable hierarchical weights under consistency control. This study aims to construct a set of Chinese HIPC curriculum evaluation system guided by the CIPP model.

## 2 Methods

### 2.1 Design

Delphi, also known as the expert consultation method, is a feedback-anonymous survey method used for qualitative analyses (19). It is a decision-making tool that enables experts with rich experience in a specific field to reach a consensus through surveys. The Delphi method enables a group to effectively solve complex problems from a collective perspective rather than an individual one. Through multiple rounds of surveys on experts’ opinions, a consensus on the research content has been reached. This method has been widely applied in multiple disciplines, playing a positive guiding role in disciplinary development and practice. It has particularly demonstrated good applicability in the field of medical education (20, 21).

**Figure 1:**
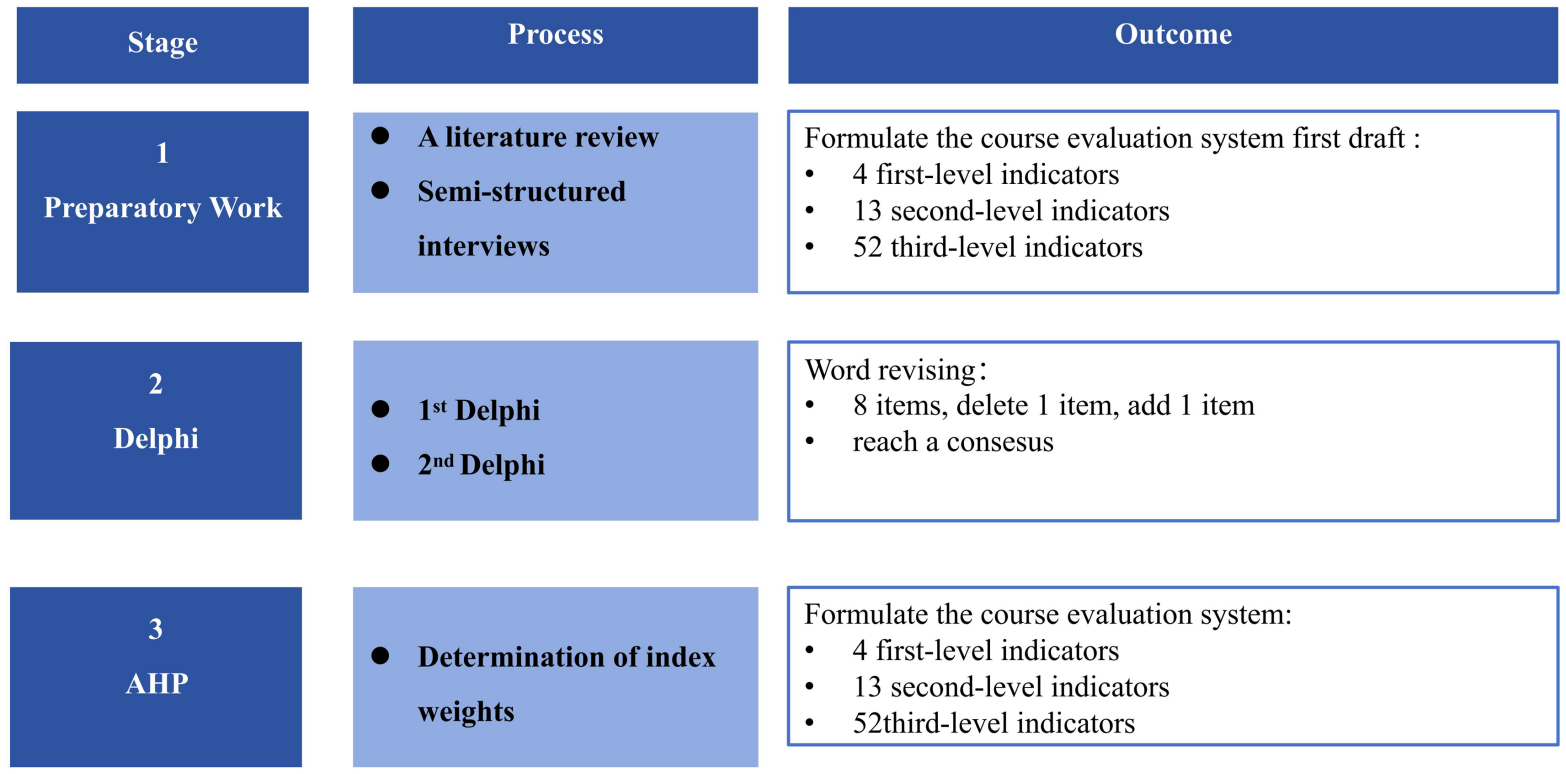
Flow chart of the research process.

This independent approach to consulting can help ensure that experts fully express their views without interference from others (22). In this study, expert opinions on the HIPC course evaluation system were collected using the Delphi method. All research steps followed the guidelines for the Conducting and Reporting of Delphi Studies (CREDES). These guidelines are presented in Supplementary Table 1. To highlight evaluation priorities and quantify evaluation indicators, we adopted the AHP for weight determination and decision-making (23). Using this method, we decomposed the decision-making problem into a hierarchical structural model, including the goal layer, criterion layer, sub-criterion layer, and so on. Then, we compared two factors within the same dimension with each other and calculated their weights. This study was conducted from February to April 2023. A total of two rounds of questionnaire consultations were carried out via email to the experts. After the conclusion of each round, the research team summarized and processed the feedback results and then distributed the questionnaire again. After the Delphi method reached a consensus, the AHP was applied to assign hierarchical weights to the indicators through pairwise comparisons and consistency checks. A flow chart illustrating the entire research process is shown in Figure 1.

### 2.2 Research team establishment

The research team assembled included one HIPC education expert and one education management expert as internal experts, and three medical graduate students. The main tasks of the team were: (1) developing the initial draft of the HIPC courses evaluation system; (2) compiling consultation questionnaires; (3) recruiting and consulting advisory experts and distributing materials; and (4) summarizing expert opinions, analyzing their suggestions, and making appropriate changes.

### 2.3 Evaluation system construction

#### 2.3.1 Initial draft

According to the CIPP model, four dimensions of HIPC courses evaluation were identified as the first-level indices, including the context evaluation, the input evaluation, the process evaluation, and the product evaluation. The context evaluation is used to diagnose the curriculum’s implementation foundation, positioning, and objectives. This dimension emphasizes the concreteness, operability, and measurability of the objectives. The input evaluation is used to examine curriculum resources, content structure, and faculty competence. The process evaluation focuses on the formative monitoring of student participation, teacher guidance, and teaching organization. The product evaluation, in turn, provides summative evidence regarding students’ experiential gains, students’ competency gains, teachers’ professional development, and the overall effectiveness of the curriculum. Fifty-nine potential indices were identified from a comprehensive literature review of Chinese databases and official documents. Then, 40 initial indices were developed through our semi-structured interviews with 6 medical educators, 6 Infection Prevention and control professionals, 6 medical staff, and 5 medical students. After internal discussion, duplicated indices were removed or merged. Finally, a preliminary advisory draft of 69 indices were formed, comprising 3 s-level and 10 third-level indices for Context evaluation, 3 s-level and 11 third-level indices for Input evaluation, 3 s-level and 15 third-level indices for Process evaluation, 4 s-level and 16 third-level indices for Product evaluation.

#### 2.3.2 Advisory questionnaire

We developed an advisory questionnaire in two rounds, comprising three parts: (1) An introduction to the contents of the questionnaire and the purpose of the research; (2) An expert consultation list including all levels of indicators of the course evaluation system for hospital infection prevention and control courses. Each indicator was evaluated using a 5-point Likert Scale, from a 5 (very important) to 1 (very unimportant) rating scale; (3) A self-evaluation form for experts, including a demographic form (age, education context, working years, professional title, and so forth.), a self-evaluation form for the familiarity with the content and a self-assessment form for reasonableness of the index judgment.

In this study, the two rounds of Delphi expert consultation questionnaires consisted of three parts: (1) Instructions for completion and research objectives, which were used to clarify the research background and answer requirements for the experts; (2) The questionnaire, which requires experts to rate the importance of each indicator in the draft evaluation system using a 5-point Likert scale, with scores ranging from 1 point (very unimportant) to 5 points (very important). (3) The expert self-assessment form, which includes demographic information (such as age, educational background, years of work experience, professional title, etc.), content familiarity, and self-assessment of indicator judgment bases on the theoretical analysis, practical experience, peer insights, and subjective self-evaluation.

### 2.4 Advisory expert identification

The Delphi expert panels in this study were drawn from various authoritative organizations in China, and the identified experts are both representative and heterogeneous. The inclusion criteria encompassed individuals who met the following qualifications: (1) possessed a minimum of 10 years of experience in clinical nursing or medical work related to infectious diseases; (2) associate senior title or above, and experience in the HIPC courses; (3) were knowledgeable about the Delphi method; and (4) expressed a willingness to participate in this study actively and complete two rounds of consultations.

### 2.5 Implementation

#### 2.5.1 First round

Members of the research team contacted the experts. After obtaining their consent, advisory questionnaires were sent to the experts by Email, and collected within 14 days. After recovering the questionnaires, we conducted statistical analyses to form a follow-up main advisory questionnaire. The selection criteria for the indicators in this study are as follows: simultaneously meeting the conditions of an importance rating mean ≥ 3.5, a full score rate > 20%, and a coefficient of variation < 0.25. The suggestions from experts regarding additions, deletions, or other modifications to the evaluation indicators at all levels were summarized and organized by the researchers, after which the project team members collectively discussed and decided whether to adopt them. After the first round, 7 indicators were revised, 4 indicators were deleted, and one indicator was added.

#### 2.5.2 Second round

In the second-round consultation, indices in the main questionnaire were modified, added, or deleted based on the results of consultation in the previous round. After modifying the main advisory questionnaire, the experts were reconsulted by Email. After two rounds of consultation, the experts’ opinions converged, and no indicators were added or removed. Only minor revisions were made to the wording of certain indicators. Ultimately, the evaluation system for the HIPC courses was finalized, consisting of 4 primary indicators, 13 secondary indicators, and 52 tertiary indicators.

#### 2.5.3 Analytic hierarchy process

Based on the results of the two rounds of Delphi consultations, a hierarchical structure model is constructed, which includes three levels: the goal level, the criteria level, and the alternatives level (24). The goal level refers to the problem to be solved, which, in this study, is the establishment of the evaluation system for HIPC courses. The criteria level refers to the factors influencing this problem, which in this study include the primary and secondary indicators. The alternatives level refers to the various solutions needed to achieve the goal, which in this study are the tertiary indicators. Then, through expert scoring or existing mean differences, the relative importance of different indicators is compared pairwise to construct the judgment matrix. The importance comparisons between pairs of indicators are conducted using the Saaty scale, based on the experts’ judgments regarding the importance differences of the indicators to determine their weight relationships.

By normalizing the judgment matrix, the maximum eigenvalue ( $\lambda_{\mathrm{MAX}}$ ) and the corresponding eigenvector (*W*) are calculated. The eigenvector reflects the importance weights of each evaluation indicator relative to its higher-level indicators. Subsequently, a consistency check is performed on the weights to verify their logical coherence. This check is typically executed using the *CR*, which mainly assesses whether the weight ranking of the indicators within the same level is logically consistent. If *CR* < 0.10, it indicates that the consistency of the judgment matrix is within an acceptable range, implying that the obtained weights are reliable. After passing the consistency check, the weights for each level of indicators in the infection control course evaluation system are finally determined by combining the weight vectors *W* from each level.

### 2.6 Data analysis

For the statistical analysis, the IBM SPSS software (version 26.0) was employed, while Microsoft Excel 2022 was utilized for the data entry by two researchers. Descriptive statistics were computed, including means, standard deviations, frequencies, and percentages. The Yaahp12.5 software was used to calculate and analyze the index weights. The effective recovery rate of the questionnaires represented the experts’ response rate. Previous studies have shown that an effective recovery rate greater than 70% indicates a high level of experts’ response enthusiasm (25). The experts’ authority level is represented by calculating composite reliability (Cr). Cr is determined by the mean values of the experts’ judgment basis coefficient (Ca) and the self-evaluated familiarity (Cs), with the formula Cr = (Ca + Cs)/2. A higher value of Cr indicates a higher level of the experts’ authority (20). A Cr value of ≥ 0.75 indicates acceptable expert reliability, while a Cr value of > 0.80 indicates a relatively high level of expert authority. The degree of coordination of experts’ opinions refers to whether there are significant discrepancies among consulting experts in their importance evaluations of indicators at all levels. It is usually represented by the coefficient of variation (CV) and Kendall’s W for each indicator (26). The CV can reflect the fluctuation in experts’ importance ratings for a specific curriculum evaluation indicator. It is expressed as the ratio of the standard deviation to the mean of the ratings for that indicator, with a general requirement that CV < 0.25. Kendall’s W and its significance test are used to determine the degree of consistency among all consulting experts in their ratings of all curriculum evaluation indicators. The value of W ranges from 0 to 1. The closer it is to 1, the more consistent the experts’ opinions are.

### 2.7 Study rigor

Before distributing the expert consultation questionnaires, the research team provided the invited experts with a detailed explanation of the research objectives and filling instructions. After the questionnaires were collected, the team first conducted a review of the completeness of the questionnaire responses and excluded any questionnaires with missing data exceeding 10%. During the data entry phase, double independent data entry and cross-verification were adopted to ensure accuracy. The analysis and handling of the opinions provided in the questionnaires were completed through collective discussions by the research team, and final decisions were made based on a combination of subjective judgments and evidence from relevant literature.

### 2.8 Ethical approval

The study proposal was reviewed and approved by the Ethics Committee of the Affiliated Hospital of Xuzhou Medical University (XYFY2023-KL041-01). All methods were performed in accordance with the Declaration of Helsinki. The participants were guaranteed anonymity and confidentiality, and voluntary participation. All audio recordings were saved in a password-protected computer, and all paper materials are stored in a locked cabinet under the supervision of designated personnel.

## 3 Result

After two rounds of Delphi consultations, the opinions of the experts converged, leading to the final definition of the evaluation system for the HIPC courses, which includes 4 primary indicators, 13 secondary indicators, and 52 tertiary indicators.

### 3.1 Indices modification

In the first round, no revisions were proposed for the primary indicators. At the secondary level, one expert recommended combining Student Experience Gains with Student Skills Gains; we did not adopt this, as they represent fundamentally different constructs, rely on different measurement approaches, and draw on distinct evidence sources. The former captures proximal, process-related effects on learners. It is typically obtained through self-reported measures, whereas the latter reflects the translation of learning into competence and is assessed by objective performance evaluations. Moreover, consistent with the CIPP model, the product evaluation explicitly addresses both proximal and terminal learning outcomes. Accordingly, we retained them as two distinct secondary indicators. At the tertiary level, input from eight experts resulted in revisions to the wording of seven items, deletion of four items, and addition of one item. These changes were made to improve clarity and operationalization by aligning statements with auditable evidence sources (25). For example, documented course design, authority-approved textbooks, and records of instructional implementation. In addition, we split the previously composite satisfaction indicator into two standalone items, including “High satisfaction with teachers’ teaching effectiveness” and “High satisfaction with the course learning experience” to avoid redundancy and enhance construct validity. The indicators that were deleted were primarily removed due to content redundancy or conceptual overlap with other indicators. The newly added item—“Teachers’ teaching abilities and attitudes”—captures a core determinant of course quality. Research indicates that instructors’ pedagogical competence and professional attitude directly influence course effectiveness and student learning outcomes (27). In designing for teaching quality, the principle of constructive alignment should be foregrounded: intended learning outcomes, teaching–learning activities, and assessment tasks must be coherently aligned, and the instructor’s pedagogical competence and professional stance in instructional design are pivotal to achieving—and sustaining—this alignment (28). By treating this indicator as an independent item, we enhance content validity and strengthen the theoretical grounding, while providing a more targeted lever for subsequent weighting and improvement. By retaining this indicator as a standalone item, we strengthen content validity and reinforce the conceptual underpinnings, while providing a more targeted lever for subsequent weighting and improvement. The finalized evaluation framework comprises 4 primary, 13 secondary, and 50 tertiary indicators. Detailed Round-1 Delphi results across all levels are presented in Table 1.

**Table 1:** Results of the first round of the Delphi survey.

Subjects	Mean	Standard deviation	Coefficient of variation	Full score rate (%)
1 Course context	4.50	0.60	0.13	55.56
1.1 Course implementation foundation	4.28	0.65	0.15	38.89
1.1.1 Students possess foundational medical knowledge related to infection control	4.44	0.60	0.13	50.00
1.1.2 Teaching equipment and programs meet the requirements for course implementation	4.39	0.76	0.17	55.56
1.1.3 Instruction tailored to students’ learning needs	4.00	0.75	0.19	22.22
1.1.4 Teachers’ interest and professional competence	4.11	1.05	0.25	44.44
1.2 Course positioning	4.72	0.56	0.12	77.78
1.2.1 A required course in medical undergraduate education that combines professional theory with quality education	4.72	0.45	0.09	72.22
1.2.2 Adherence to core values of student-centeredness, output orientation, and continuous improvement in the curriculum	4.44	0.68	0.15	55.56
1.2.3 Integration of HIPC course content with preservation of distinctive course features	4.72	0.45	0.09	72.22
1.3 Course objectives	4.89	0.31	0.06	88.89
1.3.1 Clear course objectives that are specific, actionable, and measurable, meeting national and industry needs for infection control professionals	4.94	0.23	0.05	94.44
1.3.2 Integration of teaching content with ideological and political education to foster student development	4.89	0.31	0.06	88.89
1.3.3 Emphasis on the organic integration of knowledge, skills, and qualities, aligning with students’ ability to address complex hospital infection issues in clinical practice	4.78	0.42	0.09	77.78
2 Course input	4.72	0.45	0.09	72.22
2.1 Course resources	4.39	0.59	0.13	44.44
2.1.1 Adoption of textbooks centrally compiled and officially approved by the national education authorities	4.28	0.80	0.19	44.44
2.1.2 Sufficient teaching resources, including equipment, training bases, and skill practice materials, to meet learning needs	4.61	0.49	0.11	61.11
2.1.3 Completion of the course teaching plan (e.g., lesson plans, schedules, presentations, materials, contingency plans, etc.)	4.44	0.60	0.13	50.00
2.1.4 Reasonable class sizes and appropriate student-to-teacher ratios	4.17	0.76	0.18	38.89
2.1.5 The practical training base possesses strong teaching and research capacity and extensive experience in instruction and training, and is well-equipped to meet students’ learning needs in clinical infection prevention and control	4.61	0.49	0.11	61.11
2.2 Course content structure	4.28	0.65	0.15	38.89
2.2.1 Select teaching content of varying depth and breadth based on analysis of student learning conditions	4.50	0.60	0.13	55.56
2.2.2 Well-organized course design with moderate difficulty and an appropriate workload (≤30 contact hours).	4.28	0.73	0.17	44.44
2.2.3 The course cultivation stages are clearly defined, with an organic integration of theory, experimentation, and practice, closely aligned with cultivation goals to meet social needs and students’ career development demands	4.72	0.45	0.09	72.22
2.3 Course faculty	4.61	0.49	0.11	61.11
2.3.1 Possess substantial scholarly expertise in infection prevention and control, extensive teaching experience, high professional competence, strong presentation skills, and exemplary professional ethics; hold a faculty rank of lecturer or above and a relevant master’s degree or higher	4.56	0.76	0.17	66.67
2.3.2 Select instructors with relevant disciplinary expertise for each content area, breaking down instructional silos to integrate knowledge	4.78	0.42	0.09	77.78
2.3.3 Actively engages in in-depth reflection and rigorous inquiry on HIPC teaching, demonstrates a strong commitment to pedagogical reform, proactively adopts new technologies, methods, and tools, and innovates HIPC teaching practices	4.72	0.45	0.09	72.22
3 Course process	4.06	0.78	0.19	33.33
3.1 Student participation process	4.44	0.50	0.11	44.44
3.1.1 Strictly adhere to classroom discipline, follow operational norms, and practice diligently	4.50	0.50	0.11	50.00
3.1.2 High enthusiasm for participating in infection control learning, with active interactions between teachers and students, as well as among students	4.56	0.50	0.11	55.56
3.1.3 Actively identify, raise, and solve problems under the guidance and demonstration of teachers	4.61	0.49	0.11	55.56
3.1.4 Actively integrate knowledge and methods from multiple disciplines to analyze and solve infection control problems	4.61	0.59	0.13	66.67
3.1.5 Consciously develop the ability for proactive learning and critical thinking	4.72	0.45	0.09	72.22
3.2 Teacher guidance process	4.50	0.50	0.11	50.00
3.2.1 Foster students’ learning initiative and active participation in instructional activities	4.44	0.60	0.13	50.00
3.2.2 Provide diverse forms of guidance, with well-organized and managed classroom activities, and offer appropriate and timely feedback to students	4.50	0.60	0.13	55.56
3.2.3 Break the traditional “lecture-style” teaching and silence, fostering an active classroom atmosphere	4.33	0.75	0.17	50.00
3.2.4 Student evaluations are conducted anonymously, with the anonymity of responses explicitly disclosed to students	3.61	1.01	0.28	22.22
3.3 Course organization process	4.39	0.76	0.17	55.56
3.3.1 Rigorous procedures for student grade assessment, emphasizing process evaluation and learning-outcomes assessment	4.61	0.76	0.16	77.78
3.3.2 Establish a dual-level supervision system involving the school, college, and student representatives to monitor and provide feedback on the teaching process	4.61	0.49	0.11	61.11
3.3.3 Strictly regulate and inspect teaching segments, with regular discussions and analyses of major issues in teaching	4.50	0.60	0.13	55.56
3.3.4 The teaching leader organizes unified lesson preparation to ensure that course content is not overlapping and is coherently sequenced	4.06	0.91	0.22	44.44
3.3.5 Practical learning units provide timely feedback on students’ clinical application performance	4.33	0.82	0.19	50.00
3.3.6 Conduct immediate evaluations after each class to monitor the achievement of teaching objectives	4.44	0.76	0.17	55.56
4 Course product	4.06	0.78	0.19	33.33
4.1 Student experience gains	4.56	0.50	0.11	55.56
4.1.1 Students’ awareness of hospital infection control has been strengthened	4.61	0.49	0.11	61.11
4.1.2 The sense of achievement in acquiring knowledge and skills has significantly improved	4.61	0.59	0.13	66.67
4.1.3 Significant improvement in student satisfaction with instructors’ teaching and the course	4.50	0.60	0.13	55.56
4.2 Student skills gains	4.94	0.23	0.05	94.44
4.2.1 Students have mastered knowledge related to infection control and understand relevant techniques	4.72	0.45	0.09	72.22
4.2.2 Cultivation of students’ clinical thinking, ability to identify problems, proactive thinking, problem-solving skills, and teamwork abilities	4.61	0.59	0.13	66.67
4.2.3 Cultivation of students’ reasoning, critical thinking, reflection, analysis skills, and insight into evaluation and decision-making	4.61	0.59	0.13	66.67
4.2.4 Diverse forms of academic outcomes, with clear structure and advanced, well-defined viewpoints in presentations	4.28	0.80	0.19	50.00
4.2.5 Enhanced self-awareness, concepts, and consciousness related to infection control	4.94	0.23	0.05	94.44
4.3 Teacher Professional Development	4.33	0.67	0.15	44.44
4.3.1 Ability to apply new teaching technologies and innovative teaching strategies	4.56	0.50	0.11	55.56
4.3.2 Capability for self-reflection, research, and improvement in teaching practices	4.61	0.59	0.13	66.67
4.3.3 Innovation in infection control research capabilities and academic achievements	4.22	0.79	0.19	44.44
4.3.4 Improved ability to organize classroom activities	4.33	0.58	0.13	38.89
4.4 Overall course effectiveness	4.61	0.59	0.13	66.67
4.4.1 Student satisfaction and efficiency and effectiveness of educational and research tasks for graduates	4.28	0.87	0.20	50.00
4.4.2 Innovative concepts in course development	4.50	0.60	0.13	55.56
4.4.3 Teaching strategies and course plans demonstrate significant advantages and potential for dissemination	4.28	0.80	0.19	50.00
4.4.4 Improvement in course satisfaction	4.33	0.67	0.15	44.44

### 3.2 Final evaluation system establishment

In the second round, no indicators were added or removed; only targeted wording refinements were made to a small subset of items. Following two rounds, expert ratings converged, yielding a finalized HIPC course evaluation framework comprising 4 primary, 13 secondary, and 52 tertiary indicators. In the second round, the primary indicators had mean importance ratings of 4.61 ~ 5.00, with CV = 0.00 ~ 0.11 and full-score rates = 61.11% ~ 100%; the secondary indicators had means of 4.33–4.94, CV = 0.05 ~ 0.15, and full-score rates = 38.89% ~ 94.44%; and the tertiary indicators had means of 3.83 ~ 4.89, CV = 0.06 ~ 0.23, and full-score rates = 27.78% ~ 88.89%. Detailed Round-2 Delphi results across all levels are presented in Table 2.

**Table 2:** Results of the second round of the Delphi survey.

Subjects	Mean	Standard deviation	Coefficient of variation	Full score rate (%)
1 Course context	4.61	0.49	0.11	61.11
1.1 Course implementation foundation	4.50	0.69	0.15	61.11
1.1.1 Students possess foundational medical knowledge related to infection control	4.22	0.71	0.17	38.89
1.1.2 Teaching equipment and programs meet the requirements for course implementation	4.56	0.60	0.13	61.11
1.1.3 Teaching design is based on students’ learning needs	4.56	0.50	0.11	55.56
1.1.4 Teachers’ teaching abilities and attitudes	4.72	0.45	0.10	72.22
1.2 Course positioning	4.67	0.58	0.12	72.22
1.2.1 A required course in medical undergraduate education that combines professional theory with quality education	4.56	0.60	0.13	61.11
1.2.2 Adherence to core values of student-centeredness, output orientation, and continuous improvement in the curriculum	4.33	0.75	0.17	50.00
1.2.3 Integration of infection control course content with distinct course characteristics	4.67	0.47	0.10	66.67
1.3 Course objectives	4.83	0.37	0.08	83.33
1.3.1 Clear course objectives that are specific, actionable, and measurable, meeting national and industry needs for infection control professionals	4.78	0.42	0.09	77.78
1.3.2 Integration of teaching content with ideological and political education to foster student development	4.72	0.45	0.10	72.22
1.3.3 Emphasis on the organic integration of knowledge, skills, and qualities, aligning with students’ ability to address complex hospital infection issues in clinical practice	4.72	0.45	0.10	72.22
2 Course input	4.61	0.49	0.11	61.11
2.1 Course resources	4.50	0.60	0.13	55.56
2.1.1 Use of textbooks approved by the national education administration	4.17	0.83	0.20	38.89
2.1.2 Sufficient teaching resources, including equipment, training bases, and skill practice materials, to meet learning needs	4.67	0.58	0.12	72.22
2.1.3 Completion of the course teaching plan (e.g., lesson plans, schedules, presentations, materials, contingency plans, etc.)	4.33	0.67	0.15	44.44
2.1.4 Reasonable class sizes and appropriate student-to-teacher ratios	4.17	0.69	0.17	33.33
2.1.5 Medical practice teaching bases possess strong professional capabilities and rich teaching experience to meet students’ learning needs in clinical infection control knowledge	4.56	0.76	0.17	66.67
2.2 Course content structure	4.44	0.68	0.15	55.56
2.2.1 Select teaching content of varying depth and breadth based on analysis of student learning conditions	4.44	0.68	0.15	55.56
2.2.2 The course is reasonably designed according to student learning conditions, meeting the needs of students at different levels, with an appropriate class schedule (within 30 class hours)	4.44	0.76	0.17	66.67
2.2.3 The course cultivation stages are clearly defined, with an organic integration of theory, experimentation, and practice, closely aligned with cultivation goals to meet social needs and students’ career development demands	4.78	0.42	0.09	77.78
2.3 Course faculty	4.61	0.49	0.11	61.11
2.3.1 Choose relevant professional teachers for different teaching content, breaking down teaching boundaries and integrating knowledge	4.56	0.50	0.11	55.56
2.3.2 Possess high academic proficiency in the field of infection management, with rich teaching experience, high competency, strong expressiveness, and a commendable teaching ethic	4.61	0.49	0.11	61.11
2.3.3 Hold a lecturer title or higher and possess a relevant master’s degree or higher	4.61	0.49	0.11	61.11
2.3.4 Actively engage in deep reflection and inquiry into infection control teaching, with a strong awareness of teaching reform	4.56	0.50	0.11	55.56
2.3.5Actively adopt new technologies, methods, and tools to innovate infection control teaching methods	4.56	0.50	0.11	55.56
3 Course process	5.00	0.00	0.00	100
3.1 Student Participation Process	4.56	0.50	0.11	55.56
3.1.1 Strictly adhere to classroom discipline, follow operational norms, and practice diligently	4.44	0.68	0.15	55.56
3.1.2 High enthusiasm for participating in infection control learning, with active interactions between teachers and students, as well as among students	4.61	0.59	0.13	66.67
3.1.3 Actively identify, raise, and solve problems under the guidance and demonstration of teachers	4.67	0.58	0.12	72.22
3.1.4 Actively integrate knowledge and methods from multiple disciplines to analyze and solve infection control problems	4.72	0.45	0.10	72.22
3.1.5 Consciously develop the ability for proactive learning and critical thinking	4.56	0.50	0.11	55.56
3.2 Teacher guidance process	4.61	0.49	0.11	61.11
3.2.1 Stimulate students’ interest in learning and guide their active participation in teaching activities	4.61	0.59	0.13	66.67
3.2.2 Provide diverse forms of guidance, with well-organized and managed classroom activities, and offer appropriate and timely feedback to students	4.39	0.68	0.16	55.56
3.2.3 Break the traditional “lecture-style” teaching and silence, fostering an active classroom atmosphere	4.39	0.76	0.17	66.67
3.3 Course organization process	4.44	0.68	0.15	55.56
3.3.1 Establish a dual-level supervision system involving the school, college, and student representatives to monitor and provide feedback on the teaching process	4.50	0.60	0.13	61.11
3.3.2 Strictly regulate and inspect teaching segments, with regular discussions and analyses of major issues in teaching	4.28	0.80	0.19	50.00
3.3.3 The teaching leader organizes unified lesson preparation to ensure that course content is not overlapping and is coherently sequenced	3.83	0.83	0.22	27.78
3.3.4 Practical learning units provide timely feedback on students’ clinical application performance	4.17	0.60	0.14	61.11
3.3.5 Conduct immediate evaluations after each class to monitor the achievement of teaching objectives	4.50	0.60	0.13	61.11
4 Course product	4.78	0.42	0.09	77.78
4.1 Student experience gains	4.83	0.37	0.08	83.33
4.1.1 Students’ awareness of hospital infection control has been strengthened	4.72	0.56	0.12	77.78
4.1.2 The sense of achievement in acquiring knowledge and skills has significantly improved	4.89	0.31	0.06	88.89
4.1.3 High satisfaction with teachers’ teaching effectiveness	4.61	0.49	0.11	61.11
4.1.4 High satisfaction with the course learning experience	4.67	0.58	0.12	72.22
4.2 Student skills gains	4.94	0.23	0.05	94.44
4.2.1 Students have mastered knowledge related to infection control and understand relevant techniques	4.83	0.37	0.08	83.33
4.2.2 Cultivation of students’ clinical thinking, ability to identify problems, proactive thinking, problem-solving skills, and teamwork abilities	4.78	0.53	0.11	83.33
4.2.3 Cultivation of students’ reasoning, critical thinking, reflection, analysis skills, and insight into evaluation and decision-making	4.39	0.76	0.17	66.67
4.2.4 Diverse forms of academic outcomes, with clear structure and advanced, well-defined viewpoints in presentations	4.33	0.67	0.15	44.44
4.2.5 Enhanced self-awareness, concepts, and consciousness related to infection control	4.83	0.37	0.08	83.33
4.3 Teacher professional development	4.33	0.58	0.13	38.89
4.3.1 Ability to apply new teaching technologies and innovative teaching strategies	4.22	0.79	0.19	44.44
4.3.2 Capability for self-reflection, research, and improvement in teaching practices	4.44	0.60	0.14	61.11
4.3.3 Innovation in infection control research capabilities and academic achievements	4.06	0.85	0.21	38.89
4.3.4 Improved ability to organize classroom activities	4.33	0.67	0.15	44.44
4.4 Overall course effectiveness	4.67	0.47	0.10	66.67
4.4.1 Student satisfaction and efficiency and effectiveness of educational and research tasks for graduates	4.22	0.97	0.23	44.44
4.4.2 Innovative concepts in course development	4.50	0.76	0.17	66.67
4.4.3 Teaching strategies and course plans demonstrate significant advantages and potential for dissemination	4.61	0.49	0.11	61.11

### 3.3 Basic information of the experts

This study invited 18 experts, who fully participated in both rounds of the Delphi consultation. These experts were drawn from 7 tertiary grade A hospitals (all university-affiliated) and 11 higher medical institutions located across four provinces and municipalities—Jiangsu, Beijing, Sichuan, and Taiwan. The experts came from diverse fields, including infection control management (33.33%), clinical nursing (16.67%), clinical medicine (16.67%), and infection control education (33.33%), which helped mitigate the risk of bias or limitations in the consultation results. Among the Delphi consultation experts, 7 are master’s degree (38.89%) and 6 are doctor’s degree (33.33%). With an average work experience of 25.1 ± 9.3 years and an average age of 51.4 ± 11.3 years, this expert team demonstrates extensive practical experience and profound professional knowledge in their respective fields (Table 3).

**Table 3:** Demographic characteristics of the expert panel.

Characteristics	Number (%^a^)
Gender	
Male	9(50.00)
Female	9(50.00)
Age (years)	
30~39	1(5.56)
40~49	8(44.44)
50~59	7(38.89)
60~65	2(11.11)
Educational background	
Bachelor’s degree	5(27.78)
Master’s degree	7(38.89)
Doctor’s degree	6(33.33)
Profession titles	
Senior	8(44.44)
Associate professor	10(55.56)
Professional experience (years)	
10–20	8(44.44)
21–30	5(27.78)
31–40	4(22.22)
>41	1(5.56)
Mentor type	
Master supervisor	12(66.67)
Others	6(33.33)

a Indicates the proportion of each characteristic in the total sample.

### 3.4 Degree of activeness of experts

We assessed the level of expert engagement by analyzing the effective return rate of the surveys. In each of the two rounds, 18 questionnaires were distributed, and we received 18 effective responses in both rounds, resulting in a 100% effective return rate for each round. Eight experts (constituting 44.44% of the total experts) provided their valuable opinions in the first round of the survey. In the subsequent second round, three experts (comprising 16.67% of the total experts) contributed their insights and feedback.

### 3.5 Authority coefficient of experts

In this study, the Cas in both survey rounds were 0.905 and 0.917, while the Css were 0.833 and 0.856, respectively. The Cr was 0.869 and 0.887, respectively, meeting the expert consultation authority coefficient > 0.75 standard.

### 3.6 Coordination degree of expert opinions

The degree of coordination among expert opinions was presented by calculating the CV and Kendall’s *W* (29). The CVs of both rounds of the Delphi survey were 0.050 ~ 0.230 and 0.000 ~ 0.231. The Kendall’s *W* of the indicators in both rounds were 0.153 ~ 0.162 and 0.168 ~ 0.175, respectively. We calculated the *p*-value of the first, second, and third-level indicators, which had statistical significance (*p* < 0.05) (Table 4).

**Table 4:** Expert coordination coefficients.

Items	Indicators	Kendall’s W	*χ* ^2^	*p* values
First round				
First level indicators	4	0.153	8.287	0.040
Second level indicators	13	0.162	35.084	<0.001
Third level indicators	50	0.158	145.167	<0.001
Second round				
First level indicators	4	0.175	9.429	0.024
Second level indicators	13	0.171	36.972	<0.001
Third level indicators	52	0.168	147.908	<0.001

### 3.7 Weight analysis

AHP was adopted to quantify the subjective evaluation, and experts were invited to analyze the weight of the index system to obtain the comprehensive weight value of each index in the index system, so as to judge the relative importance of each index within the same index. In this study, the criteria level refers to the primary and secondary indicators, and the protocol level refers to the tertiary indicators. After that, the judgment matrix was constructed, and the consistency was checked. When CR < 0.10, it indicates that the consistency of the judgment matrix is within the acceptable range, which means that the weight obtained is credible. The primary $\lambda_{\mathrm{MAX}}$ is 4.0206, and CR is 0.0077 (< 0.10). The first-level indicators $\lambda_{\mathrm{MAX}}$ are 3.0536, 3.0183, 3.0092, and 4.1323, respectively, corresponding to CR values of 0.0516, 0.0176, 0.0088, and 0.0496 (< 0.10). Detailed weights and combination weights are presented in Table 5.

**Table 5:** Combined weights based on the AHP method.

Subjects	Weight	Portfolio weight	CR^a^
1 Course context	0.0877	–	0.0516
1.1 Course implementation foundation	0.3108	0.0273	0.0364
1.1.1 Students possess foundational medical knowledge related to infection control	0.0670	0.0018	–
1.1.2 Teaching equipment and programs meet the requirements for course implementation	0.2095	0.0057	–
1.1.3 Teaching design is based on students’ learning needs	0.2095	0.0057	–
1.1.4 Teachers’ teaching abilities and attitudes	0.5140	0.0140	
1.2 Course positioning	0.1958	0.0172	0.0176
1.2.1 A required course in medical undergraduate education that combines professional theory with quality education	0.3196	0.0055	–
1.2.2 Adherence to core values of student-centeredness, output orientation, and continuous improvement in the curriculum	0.1220	0.0021	–
1.2.3 Integration of infection control course content with distinct course characteristics	0.5584	0.0096	–
1.3 Course objectives	0.4934	0.0433	< 0.001
1.3.1 Clear course objectives that are specific, actionable, and measurable, meeting national and industry needs for infection control professionals	0.5000	0.0216	–
1.3.2 Integration of teaching content with ideological and political education to foster student development	0.2500	0.0108	–
1.3.3 Emphasis on the organic integration of knowledge, skills, and qualities, aligning with students’ ability to address complex hospital infection issues in clinical practice	0.2500	0.0108	–
2 Course input	0.0877	–	0.0176
2.1 Course resources	0.2385	0.0209	0.0312
2.1.1 Use of textbooks approved by the national education administration	0.0660	0.0014	–
2.1.2 Sufficient teaching resources, including equipment, training bases, and skill practice materials, to meet learning needs	0.4375	0.0092	–
2.1.3 Completion of the course teaching plan (e.g., lesson plans, schedules, presentations, materials, contingency plans, etc.)	0.1451	0.0030	–
2.1.4 Reasonable class sizes and appropriate student-to-teacher ratios	0.0660	0.0014	–
2.1.5 Medical practice teaching bases possess strong professional capabilities and rich teaching experience to meet students’ learning needs in clinical infection control knowledge	0.2855	0.0060	–
2.2 Course content structure	0.1365	0.0120	< 0.001
2.2.1 Select teaching content of varying depth and breadth based on analysis of student learning conditions	0.1667	0.0020	–
2.2.2 The course is reasonably designed according to student learning conditions, meeting the needs of students at different levels, with an appropriate class schedule (within 30 class hours)	0.1667	0.0020	–
2.2.3 The course cultivation stages are clearly defined, with an organic integration of theory, experimentation, and practice, closely aligned with cultivation goals to meet social needs and students’ career development demands	0.6667	0.0080	–
2.3 Course faculty	0.6250	0.0548	< 0.001
2.3.1 Choose relevant professional teachers for different teaching content, breaking down teaching boundaries and integrating knowledge	0.1429	0.0078	–
2.3.2 Possess high academic proficiency in the field of infection management, with rich teaching experience, high competency, strong expressiveness, and a commendable teaching ethic	0.2857	0.0157	–
2.3.3 Hold a lecturer title or higher and possess a relevant master’s degree or higher	0.2857	0.0157	–
2.3.4 Actively engage in deep reflection and inquiry into infection control teaching, with a strong awareness of teaching reform	0.1429	0.0078	–
2.3.5Actively adopt new technologies, methods, and tools to innovate infection control teaching methods	0.1429	0.0078	–
3 Course process	0.5857	–	0.0088
3.1 Student participation process	0.5396	0.3161	0.0800
3.1.1 Strictly adhere to classroom discipline, follow operational norms, and practice diligently	0.1982	0.0626	–
3.1.2 High enthusiasm for participating in infection control learning, with active interactions between teachers and students, as well as among students	0.2288	0.0723	–
3.1.3 Actively identify, raise, and solve problems under the guidance and demonstration of teachers	0.3636	0.1149	–
3.1.4 Actively integrate knowledge and methods from multiple disciplines to analyze and solve infection control problems	0.1133	0.0358	–
3.1.5 Consciously develop the ability for proactive learning and critical thinking	0.0961	0.0304	–
3.2 Teacher Guidance Process	0.2970	0.1739	< 0.001
3.2.1 Stimulate students’ interest in learning and guide their active participation in teaching activities	0.6000	0.1044	–
3.2.2 Provide diverse forms of guidance, with well-organized and managed classroom activities, and offer appropriate and timely feedback to students	0.2000	0.0348	–
3.2.3 Break the traditional “lecture-style” teaching and silence, fostering an active classroom atmosphere	0.2000	0.0348	–
3.3 Course organization process	0.1634	0.0957	0.0278
3.3.1 Establish a dual-level supervision system involving the school, college, and student representatives to monitor and provide feedback on the teaching process	0.3562	0.0341	–
3.3.2 Strictly regulate and inspect teaching segments, with regular discussions and analyses of major issues in teaching	0.1513	0.0145	–
3.3.3 The teaching leader organizes unified lesson preparation to ensure that course content is not overlapping and is coherently sequenced	0.0380	0.0036	–
3.3.4 Practical learning units provide timely feedback on students’ clinical application performance	0.0983	0.0094	–
3.3.5 Conduct immediate evaluations after each class to monitor the achievement of teaching objectives	0.3562	0.0341	–
4 Course product	0.2389	–	0.0496
4.1 Student experience gains	0.2858	0.0683	0.0304
4.1.1 Students’ awareness of hospital infection control has been strengthened	0.2262	0.0154	–
4.1.2 The sense of achievement in acquiring knowledge and skills has significantly improved	0.5104	0.0348	–
4.1.3 High satisfaction with teachers’ teaching effectiveness	0.1040	0.1149	–
4.1.4 High satisfaction with the course learning experience	0.1594	0.0109	–
4.2 Student skills gains	0.5523	0.1319	0.0233
4.2.1 Students have mastered knowledge related to infection control and understand relevant techniques	0.3329	0.0439	–
4.2.2 Cultivation of students’ clinical thinking, ability to identify problems, proactive thinking, problem-solving skills, and teamwork abilities	0.2123	0.0280	–
4.2.3 Cultivation of students’ reasoning, critical thinking, reflection, analysis skills, and insight into evaluation and decision-making	0.0704	0.0093	–
4.2.4 Diverse forms of academic outcomes, with clear structure and advanced, well-defined viewpoints in presentations	0.0514	0.0068	–
4.2.5 Enhanced self-awareness, concepts, and consciousness related to infection control	0.3329	0.0439	–
4.3 Teacher Professional Development	0.0634	0.0151	0.0328
4.3.1 Ability to apply new teaching technologies and innovative teaching strategies	0.1733	0.0026	–
4.3.2 Capability for self-reflection, research, and improvement in teaching practices	0.4583	0.0069	–
4.3.3 Innovation in infection control research capabilities and academic achievements	0.0792	0.0012	–
4.3.4 Improved ability to organize classroom activities	0.2891	0.0044	–
4.4 Overall course effectiveness	0.0985	0.0235	0.0516
4.4.1 Student satisfaction and efficiency and effectiveness of educational and research tasks for graduates	0.1085	0.0026	–
4.4.2 Innovative concepts in course development	0.3445	0.0081	–
4.4.3 Teaching strategies and course plans demonstrate significant advantages and potential for dissemination	0.5469	0.0129	–

a CR, Consistency Ratio.

## 4 Discussion

### 4.1 Analysis of the scientific validity and reliability of the HIPC courses evaluation system

In this study, the Delphi method was used to construct the evaluation system of HIPC courses. The evaluation system is scientific and reliable. First of all, the experts have rich working experience in HIPC education, hospital infection management, and clinical care. In addition, the response rate was 100% in both rounds of consultation, indicating that the experts were highly motivated to treat the research. The Cr values of two rounds of Delphi were all above 0.80, indicating that the experts had a high degree of authority. The Kendall’s *W* of all indexes were statistically significant (*p* < 0.05), indicating that the results of Delphi consultation were scientific and reliable. The CR values of the indicators at all levels calculated by AHP range from 0.0000 to 0.0800 (< 0.10), indicating that the weight settings for the indicators at all levels have good consistency.

### 4.2 Analysis of specific content and weight results of course evaluation indicators

#### 4.2.1 Course context

“Course Context” in this study includes three secondary indicators: “Course Implementation Foundation,” “Course Positioning,” and “Course Objectives,” along with 10 tertiary indicators. Among these, “Course Objectives” (0.4934) and its corresponding tertiary indicator “Clear course objectives that are specific, actionable, and measurable, meeting national and industry needs for infection control professionals” have the highest combined weight, indicating that experts place the greatest emphasis on the setting of course objectives. Clear objectives are essential for the successful implementation of the course. However, universities tend to focus excessively on outcome-based approaches, overlooking the feasibility, clinical orientation, and timeliness of HIPC objectives. This may be due to the increasing emphasis on outcome-based education by education administrators (30). This study stresses that course objectives should meet clinical needs and align with societal priorities, consistent with the findings of Park et al. (31). Critically, such clarity is verifiable through syllabus review and explicit objective-assessment mapping, which reduces ambiguity at the planning and evaluation stages. “Course Implementation Foundation” ranks second in weight, with experts highlighting the importance of student needs, learning abilities, and course readiness as evidenced by documented needs assessments and a pre-course readiness checklist, such as resources, staffing, and timetabling. However, Chinese universities often do not prioritize course preparation, which may be due to an imperfect course evaluation system and uneven distribution of teaching resources. Moreover, the professional competence and teaching attitude of instructors are crucial to teaching effectiveness, consistent with the findings of Liu et al. (32). Therefore, this study recommends increased attention to the teaching ability and attitude of instructors to improve the quality of HIPC education. In practice, these aspects can be evidenced by credential or training records triangulated with structured classroom observation.

#### 4.2.2 Course input

“Course input” in this study includes three secondary indicators: “Course Resources,” “Course Content Structure,” and “Course Faculty,” along with 13 tertiary indicators. Among them, “Course Faculty” and its tertiary indicators “Possess high academic proficiency in the field of infection management, with rich teaching experience, high competency, strong expressiveness, and a commendable teaching ethic” and “Hold a lecturer title or higher and possess a relevant master’s degree or higher” have the highest combined weight, indicating that experts place great importance on the quality of the teaching team. The prominence of Course Faculty reflects the salience of teaching competence and attitudes, which can be evidenced by credential/training records and structured observation. However, course evaluations in Chinese universities do not provide specific requirements regarding the professional background, teaching experience, and ethical standards of infection control teachers.

This could be due to the fact that HIPC courses are still in the early stages of development, and the construction of infection control teaching faculty remains immature. Teachers, as the primary agents of knowledge dissemination, play a crucial role in education quality and teaching outcomes (33). The study by Muttaqin et al. (34) also confirmed that the quality of faculty development significantly affects teaching performance. “Course Resources” rank second in importance, with the tertiary indicator “Sufficient teaching resources, including equipment, training bases, and skill practice materials, to meet learning needs’ having the highest combined weight. This indicates that experts believe the adequacy of course resources significantly affects the quality of HIPC courses. However, Chinese universities often do not pay enough attention to this key factor during course evaluations. HIPC courses are highly practical disciplines, and adequate teaching resources are essential to ensure that students effectively master clinical infection control skills. Therefore, medical schools should provide ample course resources to ensure the sustainable development of medical education and to meet students’ learning needs. Walters et al. (35) also found that sufficient course resources are crucial to improving the teaching quality of HIPC courses.

#### 4.2.3 Course process

The weight results indicate that the “Course Process” holds the highest combined weight coefficient among the primary indicators, reflecting experts’ unanimous agreement on its critical importance in the overall evaluation of HIPC courses. However, while Chinese universities pay attention to course process evaluation, they often emphasize quantitative measures, primarily based on student grades and theoretical knowledge mastery, thus neglecting aspects that are not easily measurable during the course process. The course process is centered on students and is the most important part of teaching. In this study, among the secondary indicators, the weight of “Student Participation Process” is the highest, indicating that experts highly value student engagement and interaction during the course. The essence of the student participation process emphasizes a student-centered approach, promoting their comprehensive development (36). The prominent weighting of student engagement underscores engagement quality, which can be operationalized via structured classroom observation and participation logs, including attendance, contributions, and practice frequency, with problem-solving evaluated through scenario-based tasks and supplemented, as needed, by brief interviews or feedback. Among the tertiary indicators, the combination weight of “Actively identify, raise, and solve problems under the guidance and demonstration of teachers” is the highest, indicating that experts believe it is crucial to cultivate students’ ability to consciously discover, raise, and solve clinical problems in infection control teaching. However, in course evaluations, Chinese universities pay much more attention to exam scores than to students’ problem-solving abilities related to infection control, which may limit the development of students’ innovative thinking when addressing clinical issues. Therefore, this study emphasizes the need to focus more on students’ practical abilities in discovering, raising, and solving problems. The combination weight of the “Teacher Guidance Process” ranks second, with “Stimulate students’ interest in learning and guide their active participation in teaching activities” having the highest combination weight. The organization skills of infection control teachers in teaching activities are key factors affecting the teaching level and students’ mastery of knowledge. To increase student interest and participation, excellent infection control educators must master a variety of teaching strategies, such as problem-based and situational teaching, and develop their own unique teaching styles (37).

#### 4.2.4 Course product

In this study, “Course Product” which includes 4 secondary indicators and 16 tertiary indicators such as “Student Experience Gains” and “Student Skills Gains” ranks second among the primary indicators, highlighting that assessing course outcomes not only clarifies students’ learning objectives but also enhances the effective implementation of HIPC textbooks and helps teachers to organize teaching and assessing tasks (38). Among the secondary indicators, the weight for “Student Skills Gains” is the highest, followed by “Student Experience Gains,” emphasizing the importance of cultivating students’ infection control-related abilities and enhancing their learning experiences. Competence outcomes were assessed via written examinations, skills testing, simulation performance, or clinical compliance audits; experiential outcomes were measured with brief questionnaires like satisfaction, engagement and self-efficacy. We recommend interpreting objective performance metrics alongside student experience to avoid reliance on a single indicator.

In recent years, higher education evaluation has increasingly focused on student-centered learning experiences, which is consistent with the findings of Wu et al. (39). Among the tertiary indicators, “High satisfaction with teachers’ teaching effectiveness” has the highest weight, reflecting experts’ emphasis on the quality of student learning experiences and teaching interactions. Ma et al. (40) suggest that medical educators should avoid using student satisfaction as a sole measure. This study contends that student satisfaction reflects students’ subjective experiences and engagement in learning, making it an important component of the evaluation system. Additionally, a successful educational model should consider multiple factors to ensure the comprehensive achievement of course objectives, which aligns with the findings of Yeung et al. (41).

The tertiary indicator “Enhanced self-awareness, concepts, and consciousness related to infection control” has a relatively high weight, indicating experts’ attention to cultivating students’ awareness of infection control and improving their professional quality. The study suggests that HIPC teaching should help medical students establish a correct understanding of HIPC, strengthen their sense of professional ethics, and enhance their risk awareness. This is consistent with the findings of Zhao et al. (42). Conversely, the tertiary indicator “Innovation in infection control research capabilities and academic achievements” has the lowest combined weight, which may be related to the fact that HIPC courses in China are still in their early stages and are primarily offered as elective courses. Additionally, the focus on developing research capabilities is mainly directed towards graduate students. As the research capabilities of medical students gain increasing attention, this study incorporates the evaluation of teachers’ research abilities in infection control into the assessment system to promote their enhancement and encourage students to actively participate in HIPC research, thereby achieving an integration of research, teaching, and learning.

## 5 Limitations and future directions

Despite its contributions, this study has limitations. First, due to resource constraints, the expert panel was relatively small; future work should expand and stratify recruitment across more regions and institutions. In addition, in the absence of authoritative quantitative standards in China, the operationalization and measurement of indicators require further specification. Subsequent research should conduct multi-center, context-sensitive pilots to develop and validate workable scoring standards that support broader implementation.

## 6 Conclusion

In the context of increasingly prominent global public health issues and heightened attention to HIPC capabilities, the quality requirements for training courses on HIPC based on clinical work needs have also been significantly raised. This study, based on the CIPP model, integrates literature research, semi-structured interviews, the Delphi method, and the AHP to construct a scientifically valid course evaluation system. This system has broad practical significance and application value for guiding the improvement of HIPC courses’ quality, providing a practical reference for the training quality of hospital infection prevention and control personnel.
